# Multiple surface wetting events in the greater Meridiani Planum region, Mars: Evidence from valley networks within ancient cratered highlands

**DOI:** 10.1002/2016GL072259

**Published:** 2017-02-08

**Authors:** R. M. E. Williams, F. C. Chuang, D. C. Berman

**Affiliations:** 1 Planetary Science Institute, Tucson, Arizona, USA

## Abstract

Morphological characterization of valley networks in three exposures of ancient cratered highlands (N_hc1_) in the greater Meridiani Planum region yields insight into the Martian aqueous history. From our mapping, key regional differences are apparent in fine-scale valley network attributes including morphologic type, planimetric form, density, and links to candidate paleolakes. This information, combined with crater retention age (inferred exposure age), provides new details on the relative timing and nature of aqueous processes in the region. Newly identified pitted-type valley networks have morphological similarity to terrestrial pitted landforms in an evaporite setting. We interpret the pitted valley networks to reflect late-stage groundwater processes concentrated along the former fluvial conduits. Evidence from this study indicates that localized reactivation of valley networks occurred during or after exhumation of eastern N_hc1_ unit.

## 1. Introduction

Valley networks are the most common drainage feature on Mars and are frequently cited as evidence of former climate conditions conducive to sustained surface overland flow of water [e.g., *Carr and Head*, 2010]. In Viking images, valley networks were defined as negative-relief, branching features, typically a few kilometers wide, >100 m deep, and up to 200 km in length [e.g., *Mars Channel Working Group*, 1983]. The majority of these “classic” valley networks are located within low-latitude to midlatitude cratered highlands and have long been associated with the main period of widespread fluvial dissection that ceased at the Noachian-Hesperian boundary (~3.7 Ga) [*Hynek and Phillips*, 2001, 2008; *Howard et al*., 2005; *Irwin et al*., 2005; *Fassett and Head*, 2008; *Hoke and Hynek*, 2009; *Carr and Head*, 2010].

Some of the ancient cratered terrain appears to be devoid of fluvial dissection [e.g., *Hynek et al*., 2010], most notably in Arabia Terra. This is contrary to warm-wet climate models [e.g., *Wordsworth et al*., 2015] that predict significant precipitation in Arabia Terra that should have resulted in surface runoff and incision. Explanations for the “missing” valley networks include a “cold-dry” climate model where snow accumulation regions coincide with the locations of valley networks [*Wordsworth et al*., 2013], or that high infiltration capacity of some materials prevented valley networks from forming [e.g., *Carr and Malin*, 2000]. However, these explanations do not take into account the complex history of widespread burial and exhumation of the Arabia Terra region revealed in high-resolution meter-scale images [*Malin and Edgett*, 2000].

Fine-scale valley networks (typically ~100 m wide, maximum width of 500 m, <20 m relief) were first identified in Mars Global Surveyor Mars Orbiter Camera images (0.5–12 m/pixel) and include ridges interpreted as inverted channels where the surrounding substrate has been removed by differential erosion [e.g., *Edgett*, 2005; *Pain et al*., 2007]. With the recognition of these fine-scale valley network morphological types [e.g., *Williams et al*., 2013], efforts to map these features are underway to more fully document the Martian fluvial record [e.g., *Williams*, 2007; *Chuang et al*., 2016]. Recent mapping by *Davis et al*. [2016] shows an extensive network of inverted channels across Arabia Terra, filling a gap in earlier valley network maps, and illustrating a relatively high concentration of inverted channels in the equatorial greater Meridiani Planum (GMP) region (5°S–5°N, 11°W–6.5°E). The juxtaposition of negative-relief and inverted fine-scale valley network morphological types in the GMP is a unique area to assess whether this morphological diversity reflects regional differences in fluvial activity, including whether the water source was from groundwater or atmospheric precipitation.

In order to characterize the aqueous history of the GMP, we examine the fine-scale valley networks in three areas that are mapped as Noachian ancient cratered highlands (N_hc1_), the lowest exposed stratigraphic unit in the region [*Hynek and Di Achille*, 2017]. These three sites are separated by a few hundred kilometers and provide an opportunity to compare the newly mapped fine-scale valley networks to classic negative-relief valley networks [*Hynek et al*., 2010]. Our objectives in this study are to document the spatial distribution and morphological attributes of fine-scale valley networks and to reconstruct the nature and sequence of aqueous events. We present a potential terrestrial analog for interpreting the role of groundwater in generating pitted valley networks, a new valley network morphologic type identified in this study. The N_hc1_ exposures surround hematite-bearing plains where the Mars Exploration Rover (MER) *opportunity* is currently conducting surface observations, providing additional geologic context for rover observations.

## 2. Background

**Figure 1:**
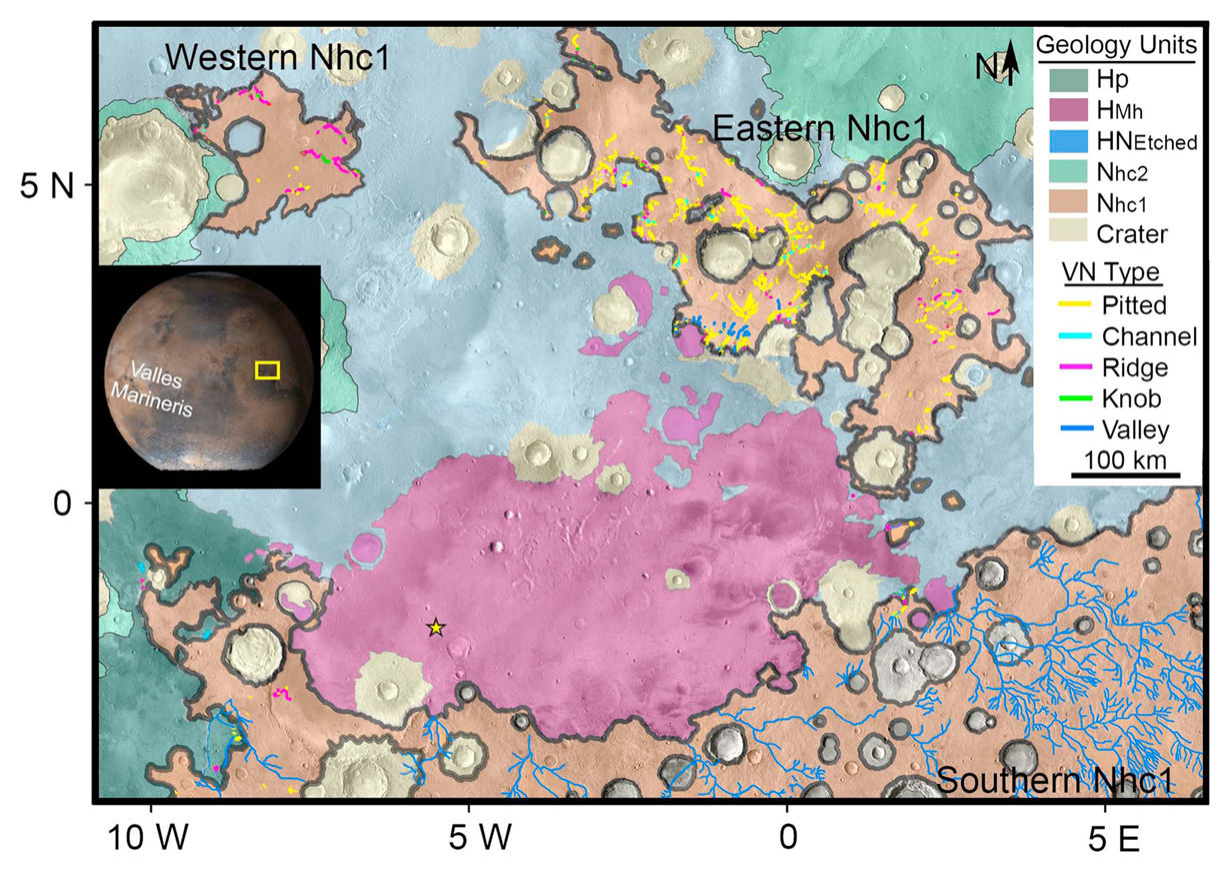
Location of fine-scale valley network (VN) morphological types in the greater Meridiani Planum study region. Geologic map units [*Hynek and Di Achille*, 2017] are overlain on a 100 m/pixel THEMIS daytime infrared mosaic basemap. From superposition relationships, basal Noachian ancient cratered terrain (N_hc1_, N_hc2_) is superposed by intermediate etched units (four subunits are grouped, blue) and uppermost Meridiani plains units (NMh, H_p_). Only valley networks mapped on three exposures of N_hc1_ (tan) are shown. Fine-scale valley network morphological types include pitted (yellow), ridge (pink), aligned knobs (green), and channels (light blue). Large-scale, negative-relief valley networks (thin dark blue) are prominent in the southern N_hc_1 unit [*Hynek et al*., 2010] and are also mapped in the eastern N_hc1_ unit (thick dark blue lines, this study). Yellow star marks the MER opportunity landing site.

Orbital data have provided abundant structural, stratigraphic, sedimentological, and mineralogical evidence for depositional events in the GMP, which is located in the southern portion of Arabia Terra (near 0°N, 0°E; Figure 1). During the Noachian Period, the Borealis impact event [*Andrews-Hanna et al*., 2008] and the tectonic load of the Tharsis region [*Phillips et al*., 2001] affected Arabia Terra. The structural uplift of the Arabia bulge produced the long-baseline topographic gradient associated with the elevation difference (hundreds of meters) among the three N_hc1_ regions (highest to the south, lowest to the northwest; Figure S1 and Table S1 in the supporting information), and the subsequent preferential fluvial transport to the northwest [*Hynek and Phillips*, 2001; *Phillips et al*., 2001; *Davis et al*., 2016].

Within the study region, there is a clear stratigraphic succession of rock units from the lowermost ancient cratered terrains to intermediate layered deposits and a capping plains unit (Figure 1) [*Hynek and Di Achille*, 2017]. The oldest unit in the region, N_hc1_, is characterized by an abundance of preserved impact craters and dark toned rock in visible wavelength images. The cratered highlands are superposed by a widespread, light toned mantling unit [*Moore*, 1990]. More recent observations have characterized this mantle unit, now referred to as “etched” terrain (HN_Etched_), as degraded layered deposits in meter-scale images [*Malin and Edgett*, 2000; *Edgett and Malin*, 2002] with low thermal inertia [*Hynek*, 2004] and sulfate mineralogy [e.g., *Gendrin et al*., 2005; *Arvidson et al*., 2005; *Wiseman et al*., 2010]. The etched deposits were once more laterally extensive with a reconstructed extent covering an area of ~3 × 10^6^ km^2^ and burying the three N_hc1_ areas by an average of ~360 m [*Zabrusky et al*., 2012]. Erosion of the etched terrain to the modern exposures occurred between ~3.83 and 3.56 Ga, bracketing the Late Noachian/Early Hesperian period [*Zabrusky et al*., 2012; *Fassett and Head*, 2007]. Multiple studies have inferred the etched materials as sulfate-rich evaporites formed in a playa setting due to fluctuating groundwater levels [e.g., *Arvidson et al*., 2006; *Wiseman et al*., 2010; *Andrews-Hanna et al*., 2007, 2010]. At the top of the stratigraphic section is a flat-lying, dark, thin Meridiani plains unit (H_Mh_) with a hematite mineralogical signature. Opportunity observations determined the hematite to be extensive lag deposits containing iron-bearing spherical concretions that eroded out from sedimentary outcrops [*Squyres et al*., 2004; *Squyres and Knoll*, 2005].

## 3. Data and Methods

The three areas of N_hc1_ in this study are based on the 1:2,000,000 scale geologic map of Meridiani Planum by *Hynek and Di Achille* [2017]. These areas are consistent with prior geomorphic studies that identified this terrain as the base of the local stratigraphic section [e.g., *Edgett*, 2005; *Arvidson et al*., 2006; *Wiseman et al*., 2010]. We refer to these units by their relative geographic position in the study area: “western,” “eastern,” and “southern” N_hc1_ (Figures 1 and S1).

Fine-scale valley networks were mapped in the eastern and western N_hc1_ units, where the range of morphological types has not been documented or studied in detail. Classic valley networks were previously mapped in the southern N_hc1_ unit [*Hynek et al*., 2010] in thermal infrared images (100 m/pixel), and we augmented these results with review of meter-scale images. Fine-scale valley networks were mapped using Mars Reconnaissance Orbiter (MRO) ConTeXt (CTX; ~6 m/pixel) [*Bell et al*., 2013] images. Where available, processed MRO High-Resolution Imaging Science Experiment (~0.25 m/pixel) [*McEwen et al*., 2007] and full-resolution Compact Reconnaissance Imaging Spectrometer for Mars (CRISM; ~18 m/pixel) [*Murchie et al*., 2007; *Michalski et al*., 2013] images were used to further assess morphology, stratigraphic relationships, and mineralogy of fine-scale valley network segments. All data sets were imported into Esri ArcGIS 10.4 software for mapping and attribution of the fine-scale valley networks.

Each segment of a fine-scale valley network was mapped as a line using ArcGIS and tagged by morphologic type (see descriptions in Text S1). For segments that appear to have continuity in forming a network system with multiple branches, a unique identification number was assigned to that system. Other information stored as attributes with each valley network segment include planimetric pattern and Strahler stream order (determined by numbering each segment, starting with the distal reaches as 1, and increasing the value by one when two equal value segments connect) [*Strahler*, 1952].

To quantify the spatial coverage of valley networks, we define regional valley network density as the ratio of total mapped valley network length to the area of the map unit [*Carr and Chuang*, 1997]. This ratio value allows for direct quantitative comparison of network density between the three N_hc1_ units. For individual valley network density (length/area), we assume that the mapped tributaries extend to the drainage divide, a conservative estimate of former extent, and use a convex-hull method [*Hoke and Hynek*, 2009] to calculate the network area. Uncertainties in accurate measurements of original valley network length and drainage basin area arise from modification of valley networks by burial and erosion. Therefore, the individual network density values reported here are only approximate to terrestrial drainage density values.

Crater counts were conducted on the western and eastern exposures of unit N_hc1_, noting those craters that clearly superpose the unit. (We did not attempt to date the valley networks themselves because of the small area and the complexities associated with burial and exhumation on crater and valley network preservation; section 5.2.) Craters greater than 100 m in diameter were mapped on CTX images, and counts excluded obvious secondary clusters. We determine the crater size-frequency populations for surface units using standard crater counting methodology as detailed in *Hartmann* [2005] and *Hartmann and Neukum* [2001]. Crater size-frequency distributions were plotted, and model absolute ages are derived using the production and chronology functions of *Hartmann* [2005] and *Michael* [2013]. Together with stratigraphic relationships, model-derived formation and exposure ages for unit N_hc1_ provide relative age constraints in the geologic reconstruction. Results are shown in supporting information (Text S2 and Figure S2).

## 4. Results

**Figure 2:**
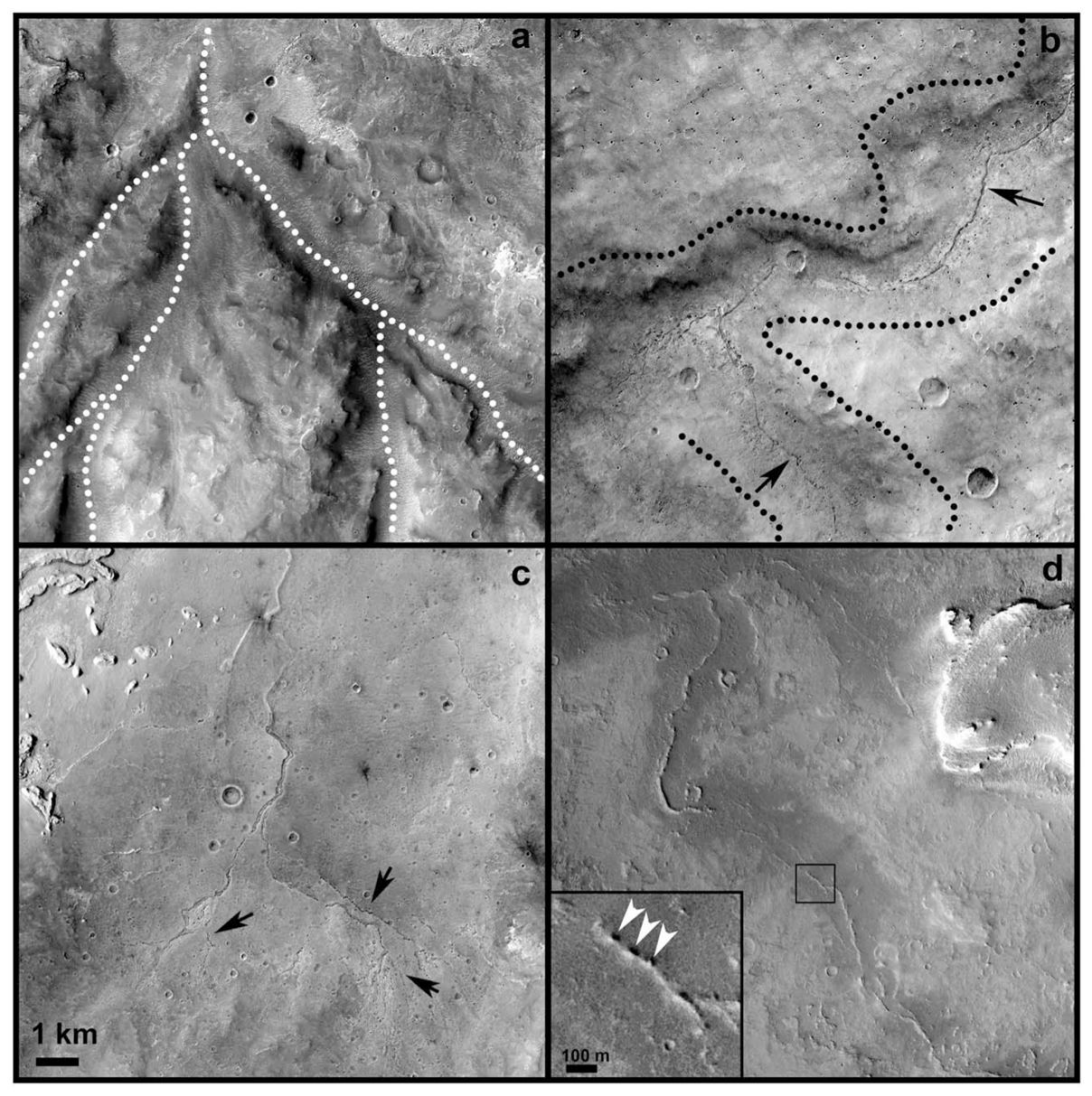
Examples of valley network morphological types in different geographic regions of ancient cratered highlands (unit N_hc1_): (a) large-scale, negative-relief valley networks dominate the southern N_hc1_ unit (white dotted line) [*Hynek et al*., 2010]; (b) a negative-relief valley network (outlined by black dotted line) with interior pits (black arrows) in the southern N_hc1_ unit; (c) pitted-type valley networks common to the eastern N_hc1_ unit, (enlargements are in Figure S3); and (d) single-thread, ridge-type valley networks, interpreted as inverted channels, are predominantly in the western N_hc1_ unit. Inset illustrates aligned knobs. Scale bar and illumination from left apply for all panels.

Four principle fine-scale valley network morphologic types are observed: pitted, channel, ridges, and knobs (Figure 2 and Text S2). Transitions between morphologic types along course are common (Figures 2c and S3). Our mapping revealed that almost all individual systems had at least two different fine-scale morphologic types, reflecting spatial variability in the factors shaping valley network types. Regardless of morphology, the fine-scale form increases in width in the inferred downstream direction (Figure S3), consistent with terrestrial river systems [*Horton*, 1945].

Pitted-type valley networks, documented for the first time in this study, are circular- to oval-shaped depressions that often intersect one another to form a chain that connects with a channel segment (Figure S2b). Pits vary in size, with the short axis length ranging from 10 to 100 m. In rare cases, aligned pits are found on the floor of classic valley networks (e.g., Figure 2b).

Compositional information for the fine-scale valley networks is sparse due to few spectral data frames, the presence of surface fines, and the small scale of the features. A limited number of processed CRISM images cover the eastern and western N_hc1_ regions, which, in general, show basaltic compositions reflecting the pervasive sand cover of this unit. Only one valley network segment, located in the eastern N_hc1_ unit, has a strong sulfate signature (Figure S4).

The fine-scale valley networks in the three regions of unit N_hc1_ differ markedly in morphologic type (Figure 1 and Table S1). In the southern N_hc1_ unit, valley networks mapped by *Hynek et al*. [2010] are large, negative-relief features and almost exclusively dendritic in planimetric pattern (Figure 2a). From our mapping of fine-scale valley networks, the eastern N_hc1_ unit has dominantly dendritic networks with pitted type, whereas the western N_hc1_ unit has mostly single-thread, ridge forms. Candidate exhumed paleolake deposits were also identified but only in the western region where circular or bulbous plateaus were observed at the termination of some ridge forms (Figure S5).

Regional differences are also apparent in the attributes of valley networks. Both eastern and southern N_hc1_ units contain the greatest number of networks and the highest stream order, some of which are space-filling branching systems comparable in scale to terrestrial river systems. In contrast, the western region has much fewer number of networks with lower stream order. Consistent with these results, regional valley network density is ~3–4 times higher in eastern and southern N_hc1_ than the western N_hc1_ region. Interestingly, individual valley network density is highest in eastern N_hc1_ (e.g., density for Figure 2c is 0.49 km^−1^), albeit over a much smaller area than in the southern N_hc1_. Pitted-type valley networks, with a greater number of closely spaced low-order segments, appear to be a more representative record of the original planimetric pattern than classic valley networks that show no microdissection (near absence of valleys <100 m wide) [*Carr and Malin*, 2000].

## 5. Discussion

Although large-scale valley networks are generally absent in eastern and western N_hc1_, the fine-scale valley networks mapped in these regions are an important record of past aqueous conditions. We interpret the fine-scale valley network morphologic types to mark the former fluvial conduit (~100 m wide). Inner channels are rarely observed for Martian valley networks because the canyon floor is typically covered by wind-blown sediment, but where seen, it is typically 90–350 m wide [*Irwin et al*., 2005], comparable in scale to the aligned pits, channels, ridges, and knobs documented here.

### 5.1. Terrestrial Analog for Martian Fine-Scale Valley Networks

To understand the processes that may have been involved in forming the fine-scale valley network morphologic types, and their interconnected configuration, documented in this study, we draw comparisons with a terrestrial analog site with denuded evaporite deposits in a hyperarid, wind-eroded setting. Features in the Pampa del Tamarugal region of the Chilean Atacama Desert have similarities to fine-scale valley networks in the GMP based on parallels in the scale and arrangement of landforms, the environment setting, and the nature of materials (composition and grain size), to the extent known.

The analog site is on a high-elevation plateau (~1000 m) situated between the Pacific coastal mountains and the higher Andes Mountains to the east. This hyperarid region receives less than 10 mm/yr of annual precipitation and almost no locally generated runoff [*Houston*, 2002; *Amundson et al*., 2012]. Hyperarid conditions in the Atacama Desert of northern Chile have existed for at least 14 million years, with periodic phases of surface flows sourced from precipitation in the Andes Mountains [*Jordan et al*., 2014]. Because of the unique environmental conditions, the region has a prevalence of hard saline crusts that are rare in other terrestrial deserts [*Stoertz and Ericksen*, 1974]. Salt crusts are formed by desiccation of episodic saline lakes [*Saez et al*., 1999], as well as by evaporation of spring water, groundwater, intermittent surface flows, and occasional floods [*Ericksen and Salas O*, 1987]. Common salt compositions include chlorides, sulfates, and borates, with anions largely derived from volcanism (e.g., atmospheric condensation from volcanic gases and hydrothermal recycling) with minor contributions from evaporated seawater transported inland via fog [*Pueyo et al*., 2001]. Similarly on Mars, multiple studies of the GMP region infer an evaporitic setting and a fluctuating groundwater table in explaining the emplacement and/or alteration of layered deposits [e.g., *Squyres and Knoll*, 2005; *Andrews-Hanna et al.*, 2007, 2010; *Wiseman et al*., 2010].

**Figure 3:**
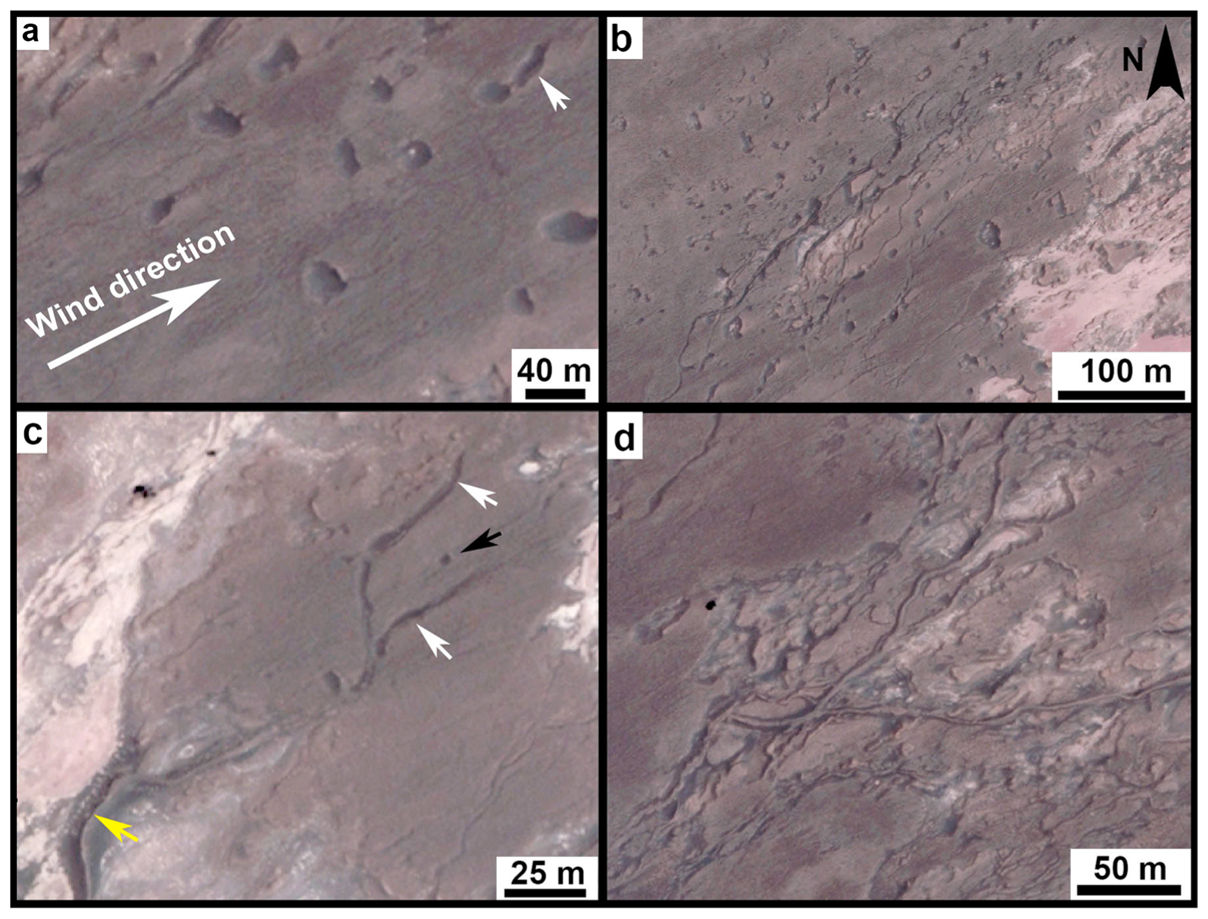
Depression and ridge morphologies formed in eroded playa sediments of the Pampa del Tamarugal region, Atacama Desert, Chile. (a) Isolated collapse pits due to dissolution of near-surface evaporites. Shallow wind-eroded grooves are black northeast-southwest streaks across scene (white arrow indicates prevailing wind direction to the northeast). (b) Circular to oblong, groundwater-fed dissolution pits are further eroded by wind scour producing scalloped terrain (surface views in Figure S7). Channels develop where groundwater flow has further dissolved surface precipitates, causing widening and eventual interconnection of adjacent pits. (c) An example of channels (white arrows) connecting to a ridge (yellow arrow), with nearby dissolution pit (black arrow). (d) Ridge network in deflated, cemented playa sediments.

In Salar de Llamara [*Quezada et al*., 2012], a complex interplay of wind and water processes has sculpted Pleistocene salt-cemented playa deposits resulting in a varied landscape of mesas, hollows, and curvilinear depressions and ridges (Figure 3). Overland surface flows including channelized flows, sheetfloods, and mudflows, as well as groundwater-surface interaction, have modified the terrain. Variability in water source and chemistry, combined with flow type and the relative timing of surface modification processes, collectively produces the diversity of landforms. We describe two formative processes associated with landforms on distal alluvial fans near Salar de Llamara (~21°S, ~69.5°W).

#### 5.1.1. The Role of Wind in Forming Ridges

Wind is a prominent force in eroding and sculpting deposits in the Atacama Desert. In Salar de Llamara, the prevailing winds (~15 m/s) are to the northeast [*Shaw and Wagner*, 2003]. Wind deflates inactive distributary channels on the distal alluvial fan, removing loose, fine-grain deposits and leaving behind a gravel lag or cemented surface forming inverted channels 3–10 m wide and 1–2 m in relief [*Morgan et al*., 2014; *Williams et al*., 2016]. Most inverted channels are curvilinear, but some exhibit high sinuosity (Figure S6).

Many ridge forms in GMP are inferred to be inverted channels where clast armoring and/or cementation protected fluvial deposits from erosion presumed to be dominantly aeolian deflation [*Davis et al*., 2016]. In general, we agree with this interpretation for ridges mapped in this study, most of which are located in western N_hc1._ These ridges are continuous over several kilometers and have few branching segments, consistent with terrestrial inverted channels which generally lack tributaries [e.g., *Williams et al*., 2011]. In addition, many ridge-type valley networks in western N_hc1_ have continuity relationships with candidate paleolake deposits (Figure S5), bolstering the interpretation of flowing and ponded water aiding preferential preservation of these deposits from erosion via cementation or fluvially transported clasts.

#### 5.1.2. The Role of Water in Forming Pits, Channels, and Ridges

In Salar de Llamara, landforms generated as a result of dissolution of salt-cemented lacustrine deposits include pitted, channel, and ridge forms with striking similarity to Martian fine-scale valley network morphologic types in GMP. Localized groundwater upwelling and associated salt dissolution result in collapse structures, which are typically circular pits (Figure 3a) [*Stoertz and Ericksen*, 1974]. With groundwater table fluctuations over time, pits enlarge and coalesce to form an irregular outline (Figure S7a) or pit chains (Figures 3b and S8a). Wind scour exploits the exposed, uncemented playa sediments, further eroding the depressions to create a scalloped terrain.

Pit chains transition downslope to curvilinear depressions and channels (Figures 3b and S8a), possibly due to dissolution associated with subsurface groundwater flow. Figure 3c illustrates of two channel-like forms converging into a single curvilinear depression which further transitions to a positive-relief ridge. This suite of landforms can result from dissolved salts that are precipitated downslope along water flow paths. Such a mechanism of solute mobilization creating voids and armoring surfaces along flow paths could also explain medial ridges within depressions (Figure S8a). Alternatively, some of the curvilinear depressions could result from surface flows where spatial variability in cements (and by extension the infiltration capacity of surface sediments) could explain the abrupt onset of incision and discontinuous channel pattern. Differentiating the relative role of subsurface groundwater flows versus overland channelized flow in creating both positive- and negative-relief curvilinear forms is difficult to distinguish based solely on morphology. Nevertheless, these examples from an evaporitic setting strongly demonstrate the influence of aqueous processes in the development of these juxtaposed positive- and negative-relief forms.

We speculate that the pitted-type valley networks in eastern N_hc1_ formed via groundwater dissolution collapse confined to the former flow conduit (e.g., inner channels) (Figures 2b and S8b). Pitted-type valley networks are steep-sided depressions with a curvilinear arrangement consistent with a structurally controlled undermining process. Individual valley networks have segments that are pitted type connected with ridges and channels, a relationship of landforms that suggest similar formation processes to those that operated in Salar de Llamara. In addition, the apparent compositional substrate where pitted-type valley networks are located, a sequence of sulfate-rich materials (etched units) covered by loose basaltic sand (Figure S4b), is similar to the evaporitic crusts susceptible to dissolution processes in the Atacama Desert. The shape and spatial distribution of pits contrasts with voids generated from surface erosion processes, such as deflation hollows that typically create shallow saucer-shaped depressions [e.g., *Hugenholtz and Wolfe*, 2006].

### 5.2. Valley Network Activity in Greater Merdiani Planum

Using the development sequence of GMP from prior studies, the mapped regional attributes of fine-scale valley networks in this study, and the Salar de Llamara analog, we discuss the nature and relative timing of aqueous processes associated with valley networks on the ancient cratered terrain. The overwhelming majority of classic valley networks in Arabia Terra appear to have been carved by the Noachian-Hesperian boundary (~3.7 Ga) [*Fassett and Head*, 2008; *Hoke and Hynek*, 2009], and we infer that surface incision via surface overland flow occurred on all N_hc1_ units in our study area by this time. However, from the morphological attributes of fine-scale valley networks, the pattern of fluvial dissection appears to differ regionally, possibly reflecting the availability of water [e.g., *Hoke and Hynek*, 2009] and/or the topographic setting. Dendritic fluvial incision was concentrated in eastern and southern N_hc1_, while the lower elevation and shallow gradient setting of western N_hc1_ was a local sedimentary sink with single-thread fluvial systems and ponded water.

Near the time of the Noachian-Hesperian boundary (~3.7 Ga), the GMP was buried by layered sedimentary materials forming the etched units (HN_Etched_; see section 2). Fluctuating water tables resulted in sulfate precipitation with thicknesses >50 m in areas [e.g., *Wiseman et al*., 2010]. Regional erosion stripped away much of the etched units by ~3.5 Ga [*Zabrusky et al*., 2012], and we speculate that groundwater dissolution processes may have been required to liberate these sulfate-cemented materials for transport analogous to the eroded playa deposits of the Atacama Desert. Although mapped as ancient cratered terrain, it is possible that eastern and western regions retain a thin veneer of etched deposits, as reflected in the younger retention ages (~3.3–3.4 Ga) from medium crater counts (Text S1 and Figures S2a and 2b), but that the landscape characteristics are consistent with exposures of N_hc1_ at mapping scale [*Hynek and Di Achille*, 2017]. Thus, some of the fine-scale valley networks mapped in this study may be within lowermost etched deposits.

We propose that the regional differences in observed valley network morphologic types, both in negative and positive relief, are closely tied to the mobility of solutes and reflect temporal changes in aqueous processes. Surface deflation in western N_hc1_ resulted in inverted fluvial systems, likely due to cementation of channel floor sediments, although no chemical signatures to support this contention have yet been observed. We infer that this landscape inversion occurred prior to deposition of the etched units based on superposition relationships (e.g., Figures S3c and S4b).

In contrast, the valley network types in eastern N_hc1_ are linked to the sulfate-rich overburden (etched terrains) and therefore postdates both (1) the majority of regional exhumation and (2) formation of the capping hematite-bearing Meridiani plains (opportunity field site). Reactivation of the former valley network conduits by groundwater-fed dissolution/precipitation processes generated pit chains, curvilinear depressions, and ridges that are analogous to those observed in Salar de Llamar, Chile. Localized reactivation of valley networks likely occurred during or after exhumation of eastern N_hc1_ unit. Exposure ages of the N_hc1_ units constrain the timing of these late-stage groundwater processes (Text S2 and Figure S2).

Results from this study indicate that surface conditions in Arabia Terra must have been warm enough to support liquid water, at least at times, in the Hesperian or later. This is consistent with previous studies that have identified aqueous activity that postdates regional widespread fluvial incision in Arabia Terra: deposition of hydrates following erosion of the etched deposits [*Wiseman et al*., 2010], a paleolake with maximum age of 3.6 Ga [*Hynek et al*., 2015], and fluvial activity in fresh shallow valleys between 3.5 and 2 Ga [*Wilson et al*., 2016]. Also, hydrological models of groundwater flow driven by precipitation, evaporation, and surface topography predict that the GMP region was a location of groundwater upwelling [*Andrews-Hanna et al*., 2010].

In conclusion, this study provides details on the history of GMP valley networks: an early phase of sustained surface overland flow, followed by a period dominated by groundwater processes associated with widespread evaporite deposition and ultimately transitioned to localized dissolution of solutes that reactivated ancient valley networks. Further constraining the relative timing and duration of these aqueous periods is of fundamental importance to an accurate characterization of past climate conditions on Mars [*Hynek*, 2016].

## Supplementary Material



## Key Points

- Mapped morphologic types of valley networks near Meridiani Planum, Mars, include channel, ridge, knob, and newly described pitted types
- Fine-scale valley networks in the study region share similar attributes with dissolution landforms in Chilean Atacama Desert
- Localized reactivation of Martian valley networks via groundwater processes occurred during or after exhumation of eastern N_hc1_ unit
