# Supplementary material for: Multiple surface wetting events in the greater Meridiani Planum region, Mars: Evidence from valley networks within ancient cratered highlands
Source: Geophys Res Lett. Author manuscript; Available in PMC 2021 Oct 12. (PMC8507182; doi:10.1002/2016GL072259)
Supplement: Supplementary file 1 [file NIHMS898392-supplement-supplement_1.pdf]

Geophysical Research Letters

Supporting Information for

Multi-Stage Surface Wetting Events in the Greater Meridiani Planum Region, Mars: Evidence from Valley Networks within Ancient Cratered Highlands

R. M. E. Williams[1], F. C. Chuang[1], and D. C. Berman[1]

[1]Planetary Science Institute, 1700 East Fort Lowell, Suite 106, Tucson, AZ 85719

Contents of this file

Text S1 to S2
Figures S1 to S8
Table S1 to S2

Introduction

This supporting information document includes additional illustrations of fine scale valley networks on Mars and examples from a terrestrial analog in the Chilean Atacama Desert. A description of the attributes associated with the four fine-scale valley network morphologic types is presented in text S1. Estimates of formation age and exposure age from crater counts are presented in Text S2 and Figure S2. Table S1 summarizes morphological attributes of valley networks in three areas of ancient cratered terrain. Source image and location information for all figures in the manuscript is documented in Table S2.

S1. Description of fine-scale valley network morphologic types

In this study, we compare fine-scale valley network morphologic types to 'classic' valley networks (Figure 2). 'Classic' valley networks are negative-relief, branching features, typically a few kilometers wide, >100 m deep, and up to 200 km in length [e.g., Mars Channel Working Group, 1983; Carr and

Chuang, 1997]. Fine-scale valley networks are typically ~100 m wide (maximum width 500 m) and four principal morphologic types are identified within the study region.

Channels. Channels are linear to sinuous negative-relief features >100 m long. Their cross-sectional forms range from shallow-wide to deep-narrow with a flat or convex floor.

Pitted. Pitted type are aligned circular to oval shaped features with the short axis ranging from 10 to 100 m (Figures 2b, 2c and S3). Pits can be singular features, but often intersect and combine with adjacent pits to form chains. Figure S3d illustrates an individual valley network with aligned pits and channel type segments.

Ridges. Ridges are positive-relief features that appear flat-topped or rounded in cross-section (Figure 2d). These forms are interpreted as inverted channels.

Knobs. Knobs are hills with flat or rounded tops found in association with ridges. Their sizes can vary, but are generally not wider than the original ridge feature. These forms are interpreted as eroded remnants of ridges (Figure 2d).

S2. Formation and exposure age estimates

Crater counts were performed on CTX images in the western and eastern N_{hc1} units (Figure S2) to determine if they are similar in age, and to compare with the ~3.92 Ga age for unit N_{hc1} reported by Hynek and Di Achille [2017]. Crater size-frequency distributions (CSFD) were plotted in differential format using Craterstats 2 software, and model absolute ages are derived using the production and chronology functions of Hartmann [2005] and Michael [2013]. The crater count statistics are robust as the areas assessed are larger than the 10,000 km^2 recommended size for age assessment using CTX data [Warner et al., 2015].

Interpretation of the formation age is difficult due to the complex history of burial and exhumation in the region; this is reflected in the crater counts, with larger craters revealing older ages than medium size craters. From the crater size-frequency distribution of all craters, both N_{hc1} exposures match the slope of the production function at medium sizes (~1 - ~8 km), giving a crater retention age of ~3.3 – 3.4 Ga. At larger bin sizes (>~8km) data points lie on older isochons; however, their error bars are large due to small statistics and may, in some cases, overlap the 3.3-3.4 Ga isochrones. Therefore, we conclude that the formation of unit N_{hc1} had occurred by at least 3.3 Ga, but may extend back to the Late Noachian. This result is consistent with the timing of deposition for the etched unit (~3.8 Ga) determined by Zabrusky et al. [2012], which occurred after emplacement of unit N_{hc1}.

For superposed craters, which likely represent the age at which both units may have been exposed, the density of craters on the eastern N_{hc1} unit is consistently lower than the western N_{hc1} unit at all diameter sizes. The associated isochron fits for craters between ~1.5 and ~4 km in diameter yield an exposure age of the eastern N_{hc1} unit that is younger (~2.1 Ga) than the western N_{hc1} unit (~2.9 Ga) for medium sized craters, although given the errors they may be separated by only a few hundred million years. Although their error bars overlap, the lower SFD density for the eastern unit is consistent over all crater size bins, suggesting the difference in exposure age between the two units is real. Rollover of the distribution occurs at crater sizes of <~1 km for both the western and eastern N_{hc1} units. However, it is steeper for the eastern N_{hc1} unit, indicating more prolonged removal of small craters in this area. We interpret the difference in exposure age between the two regions to reflect greater burial and/or erosion of the eastern N_{hc1} relative to western N_{hc1} unit. Finally, the exposure age

for western and eastern N_{hc1} units suggest that erosion of these regions may have continued into the Early Amazonian, with most of the exhumation ending at ~2.9 Ga and ~2.1 Ga, respectively.

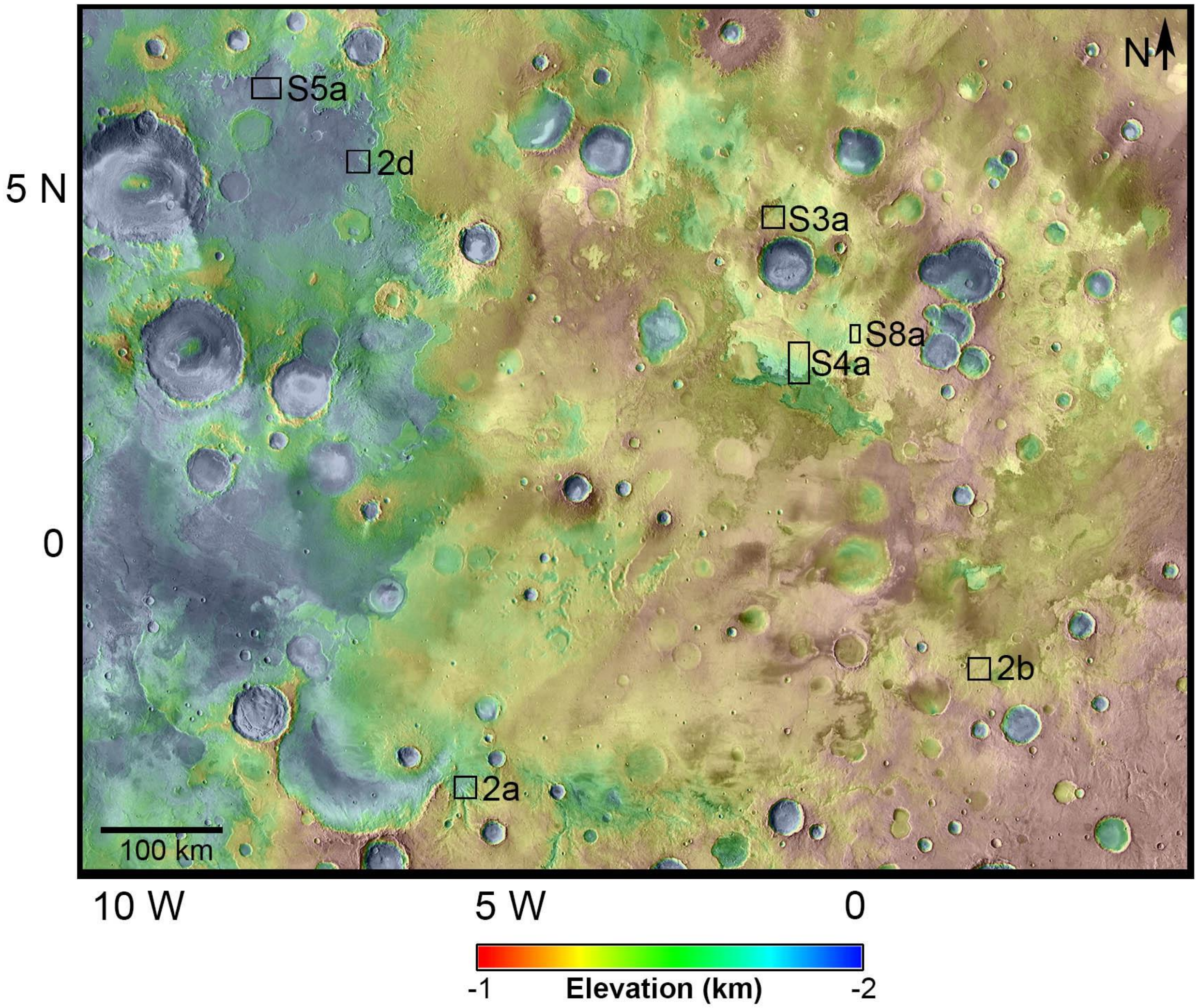

Figure S1: MOLA gridded topography (128 pixel/degree) overlain on THEMIS daytime infrared mosaic basemap for the study region with the location of figures marked. Regional topographic gradient slopes to the northwest. See Table S2 for image ID and location information.

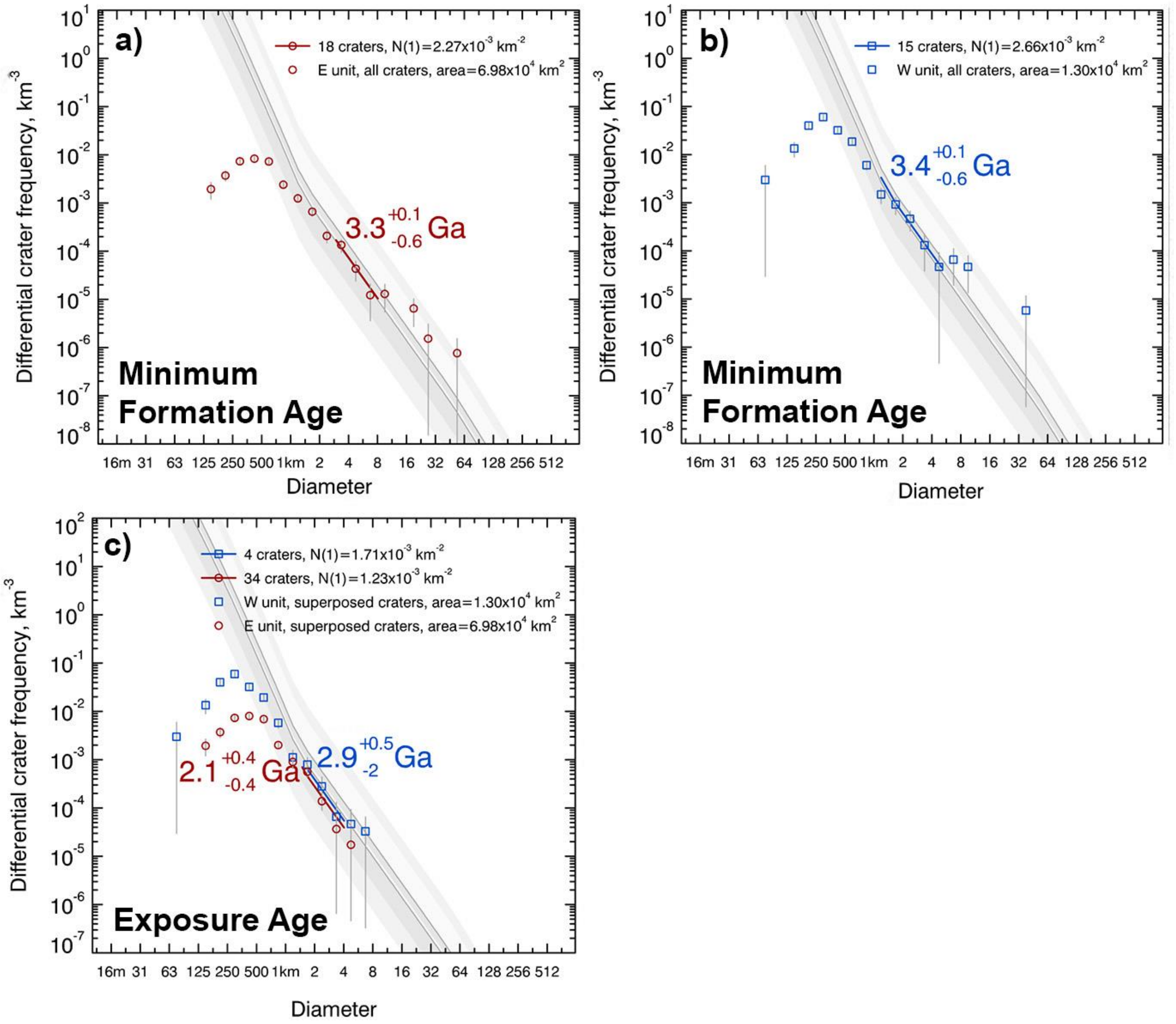

Figure S2: Crater size-frequency distribution plots for the ancient cratered terrain in the study region with all craters (superposed and older exhumed) plotted for **a)** the eastern N_{hc1} unit (red circles, **b)** the western N_{hc1} (blue squares), and **c)** only superposed craters plotted for both regions. Absolute ages are derived using the using the production and chronology functions of Hartmann [2005] and Michael [2013]. Shaded gray areas represent various periods of Martian time (e.g., early Noachian, middle Noachian, etc).

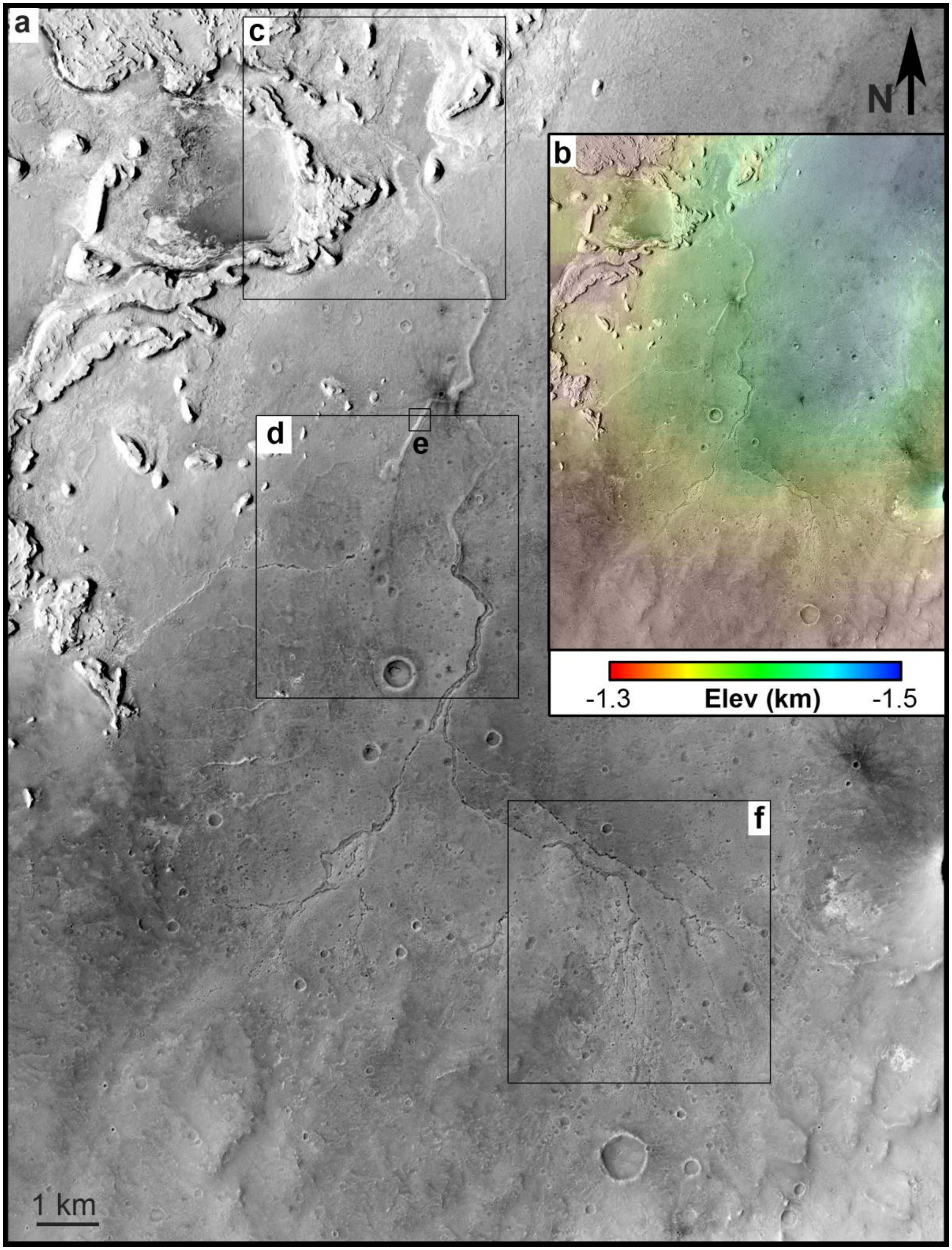
a
c
N
b
d
e
-1.3
Elev (km)
-1.5
f
1 km

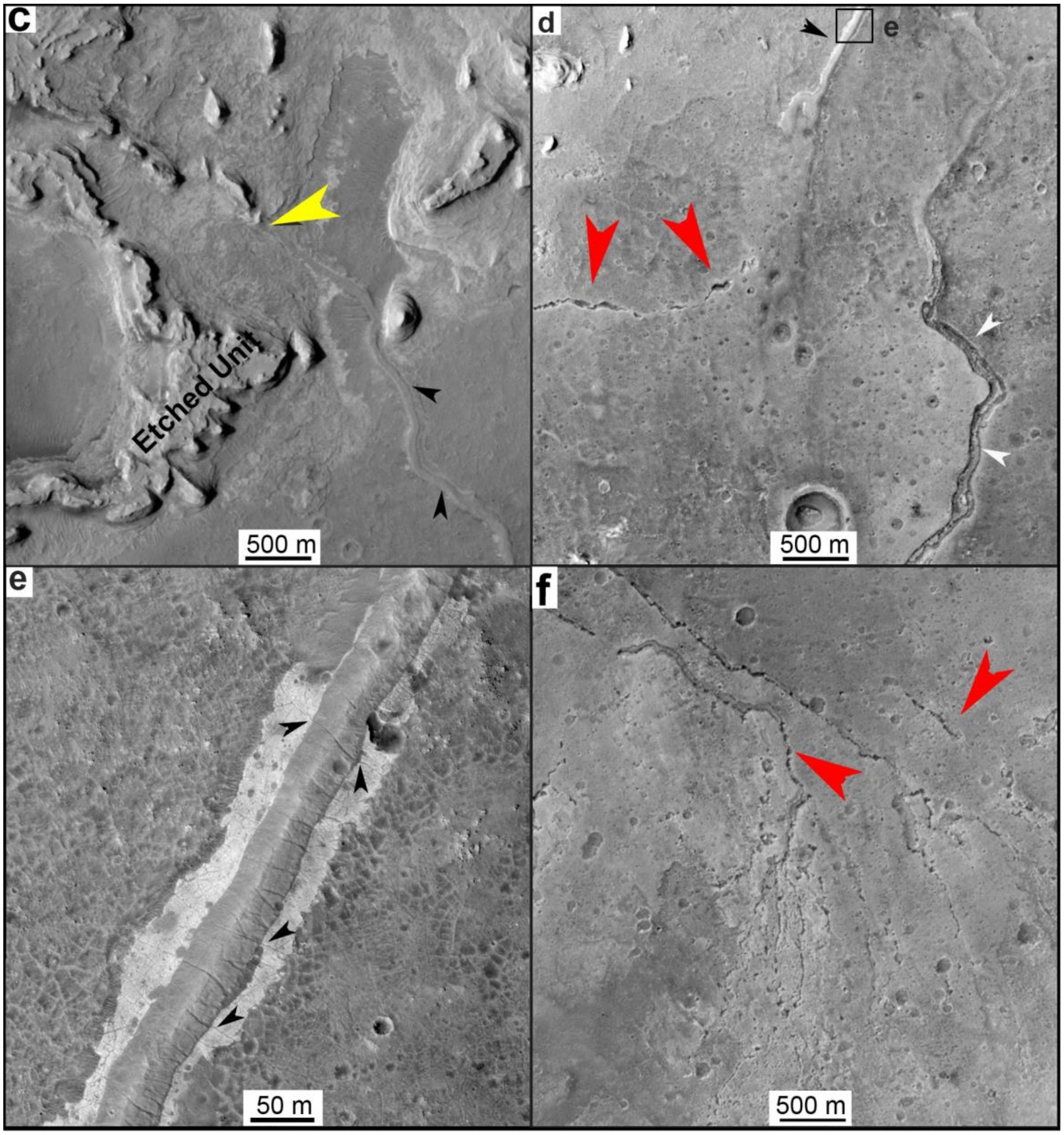

Figure S3: Examples of various preservation states in a single valley network system (same location as figure 2c). **a)** The valley network has segments in positive and negative relief. Detailed views of the morphology along reaches are illustrated in panels S3c-f. **b)** MOLA gridded topography (~463 m/pixel) for area covered in panel a. **c)** Low-relief ridge (black arrows), and location where ridge is buried by etched unit (yellow arrow). This relationship indicates that landscape inversion occurred before deposition of the etched unit. **d)** Transition of low-relief ridge (black arrows), from positive relief to negative relief with a medial ridge in places (channel, white arrows), and aligned pits (red arrows). **e)** Enlargement of two-toned ridge (black arrows) with cracks traversing across both materials (note difference in size and configuration of cracks from the polygonal pattern on the surrounding plains). **f)** At the southern end, channels and aligned pits trace out a space-filling, branching pattern. The scale of the feature grows in size in the inferred downstream direction (north) from <50 m pits (d) to ~100 m channel and ridge segments (b and c), a pattern also observed in terrestrial river systems. Illumination is from left for all panels. See Table S2 for source image information.

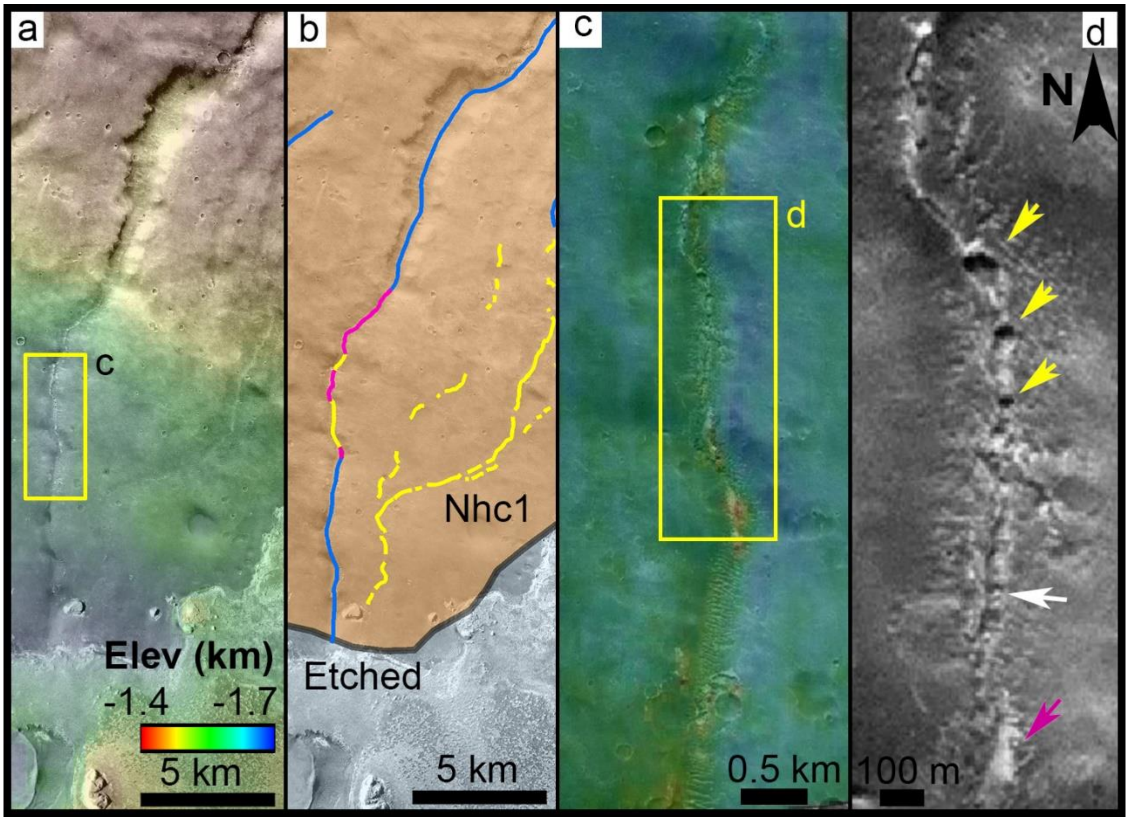

Figure S4: One newly-mapped valley network in eastern N_{hc1} has sulfate spectral signatures associated with light-toned exposures in ridge-type and pitted-type segments: **a)** CTX image, **b)** mapped valley network morphology (see legend in Figure 1), and **c)** strong sulfate signature (red). CRISM sulfate index map (R2240-R2540) with the relative intensity of spectral signature ranging from low in blue to high in red. **d)** Subscene highlighting valley network morphological types from pits (yellow arrows) to coalesced pits (white arrow) to ridge (pink arrow) with sulfate signature. See Table S2 for source image and location information.

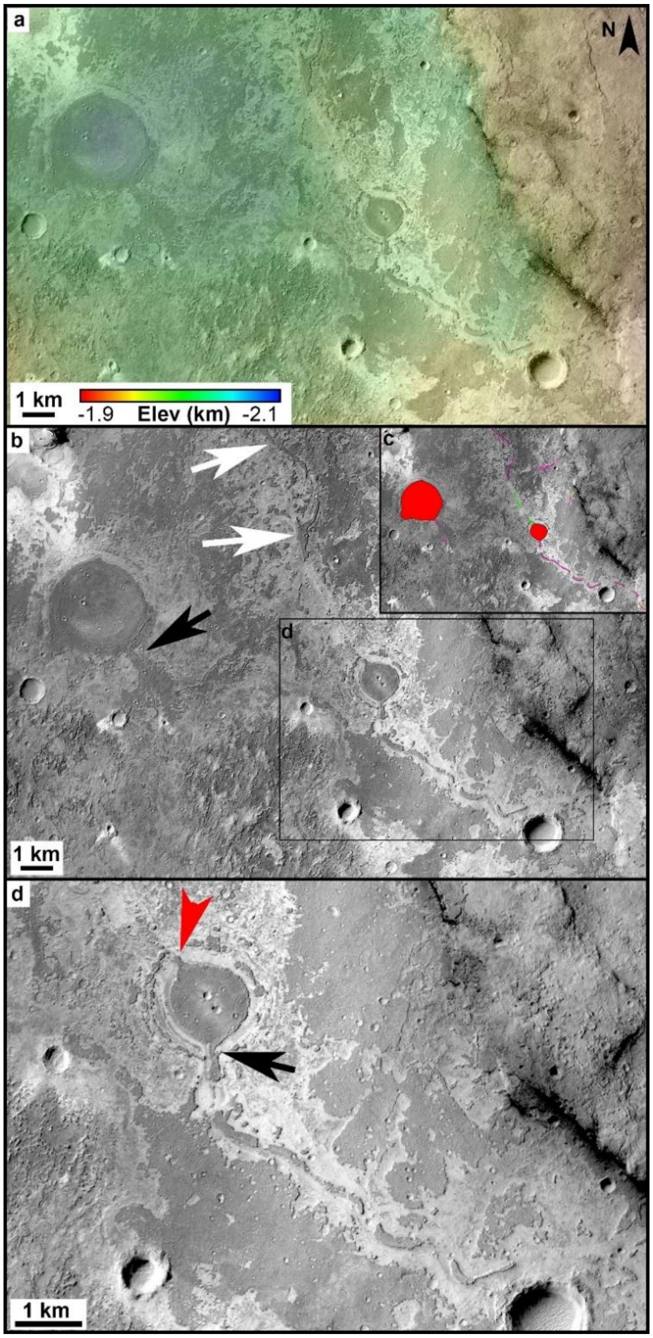

Figure S5: Candidate exhumed paleolake deposits associated with ridge forms in western N_{hc1}. **a)** MOLA gridded topographic data overlain on CTX basemap showing regional gradient to the northeast. **b)** Dark-toned circular mesas with connecting ridges may be remnant paleolake and fluvial deposits that once filled craters and channels, and are now high-standing features due to differential erosion [see also Edgett, 2005, Fassett and Head, 2007]. White arrows indicate continued path of ridge from a paleolake outlet. **c)** Map of valley network morphologic types with ridges in pink (inferred inverted channel segments of valley networks) and aligned knobs in green. Circular mesa, inferred paleolake deposits, are shown in red. **d)** Enlargement of a region in panel b with a circular mesa and associated ridge. A valley network appears to have breached into the circular basin forming an inlet (black arrow; also see panel b) with a potential outlet at another location (red arrow).

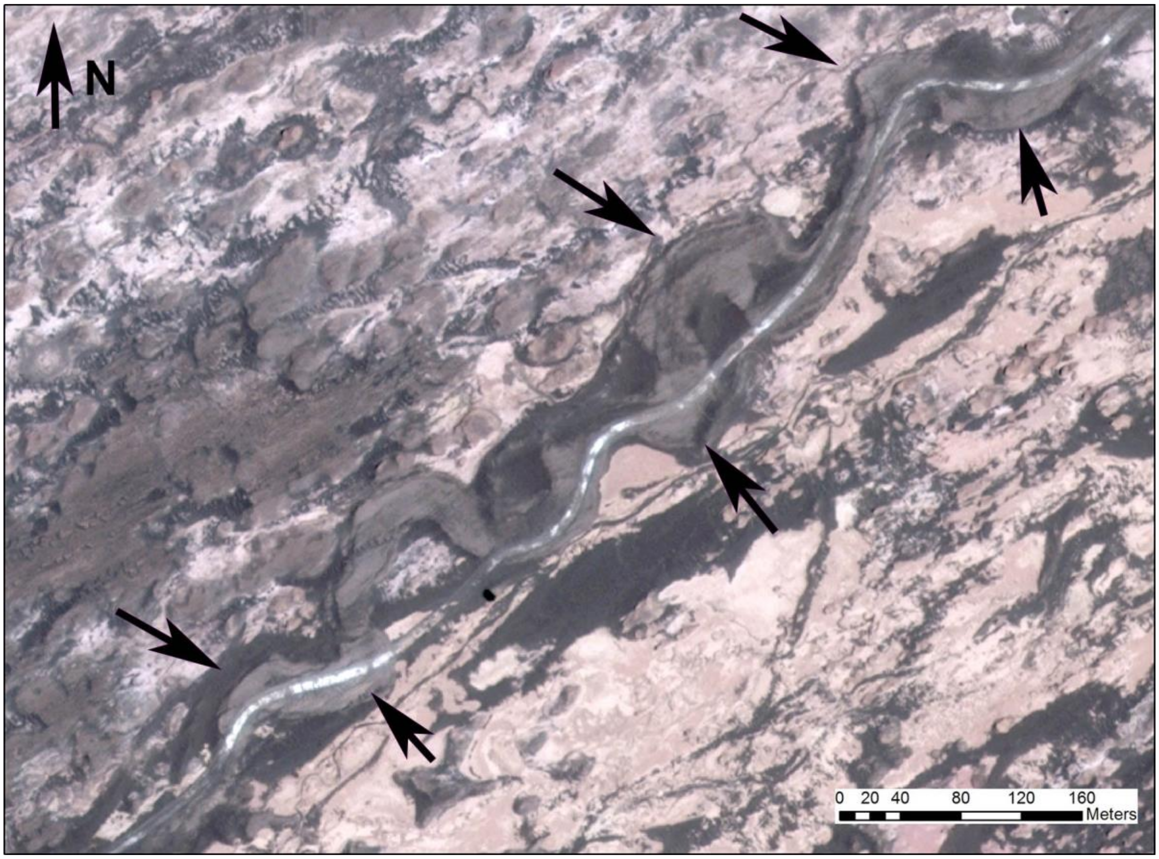

Figure S6: A sinuous inverted channel (black arrows) that formed in brown Pleistocene playa deposits of Salar de Llamara. White path is a road. See Table S2 for source image and location information.

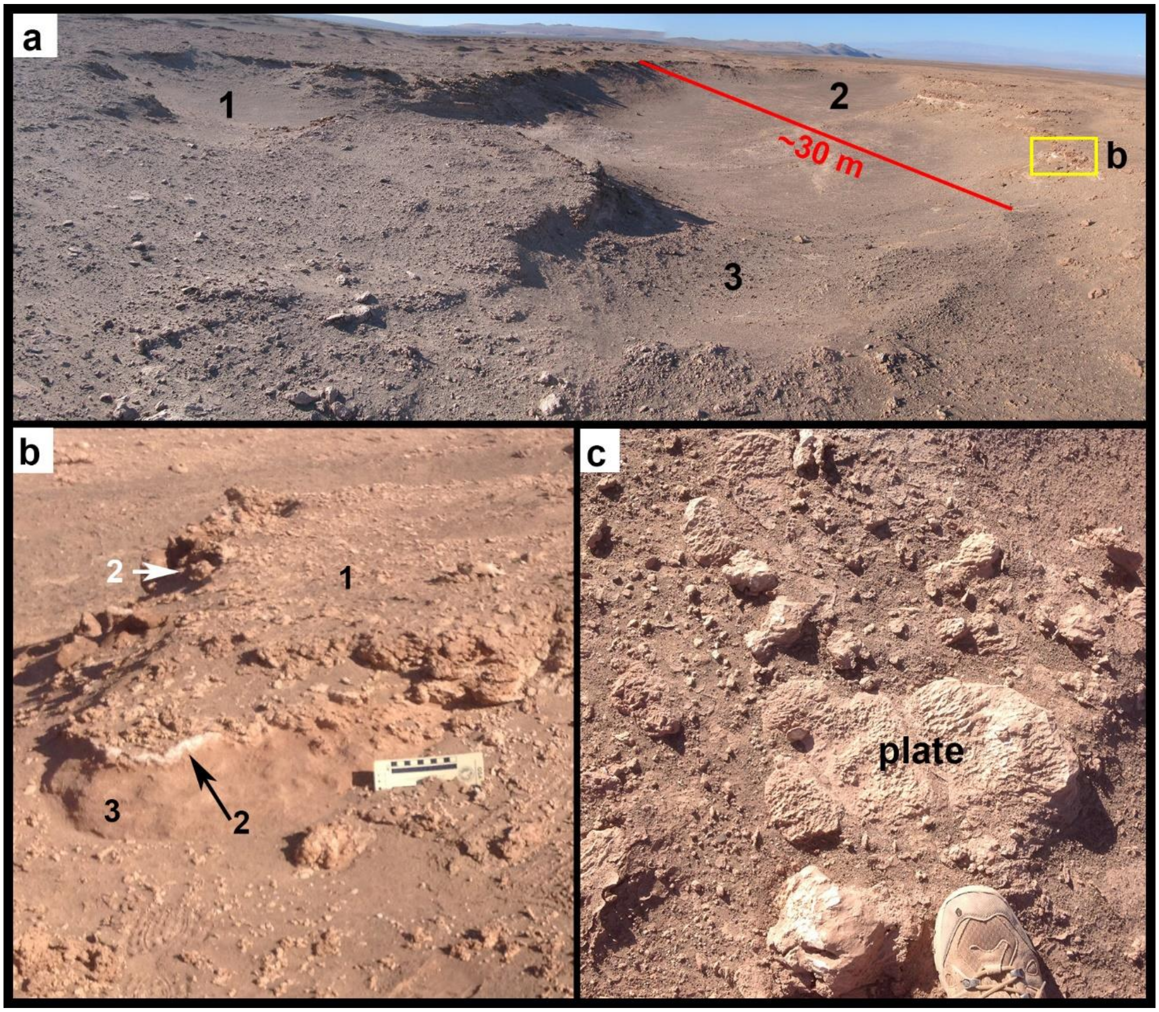

Figure S7: Examples of scalloped terrain associated with eroded playa sediments in the Pampa del Tamarugal region of the Atacama Desert, Chile. **a)** Combined dissolution and wind scour of evaporite-crusted playa sediments produces coalesced pits (1-3). **b)** Playa surface crust with three zones: (1) upper calcium sulfate crust, (2) halite horizon (white band and nodules), and (3) uncemented, fine-grained lacustrine sediments. Scale bar is 10 cm. **c)** Playa sediments capped by discontinuous, thin (<15 cm) calcium sulfate plates.

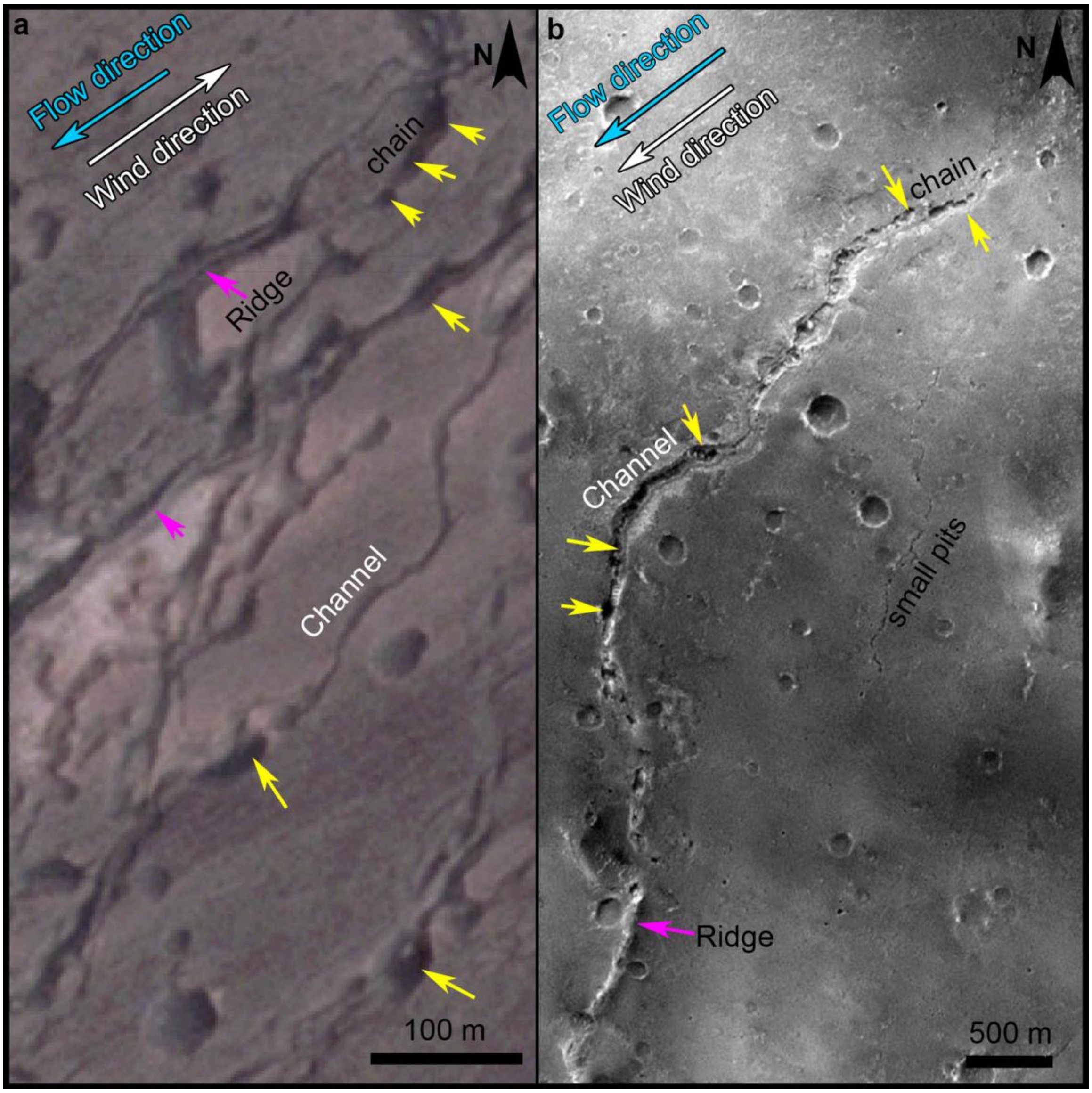

Figure S8: Comparison of landforms in (**a**) the Pampa del Tamarugal region of the Atacama Desert, Chile to (b) the greater Meridiani Planum (GMP) region on Mars. Dissolution generates pits (yellow arrows), some of which are aligned in chains or coalesce into curvilinear troughs due to groundwater flow and/or wind erosion (blue arrow marks prevailing wind direction). Ridges marks former fluvial paths that are now preserved as inverted relief features. Compare medial ridge in panel **a** to panel S3d. Similar to the Atacama pitted terrain, eolian activity in GMP may further erode the pits, especially in the southwest direction that is reflected in modern wind streaks. See Table S2 for source image information.

Table S1: Morphologic Attributes of Aqueous Landforms in N_{hc1}

Attribute	Southern N_{hc1}	Western N_{hc1}	Eastern N_{hc1}
Area[1] (km^2)	132,130	13,009	61,434
Elevation Range (km)	--2.2 to -1.0	-2.0 to -1.9 m	-1.30 to -1.0
# of Networks	25	5	91
Dominant VN type	Large-scale, Negative Relief	Fine-scale, Ridge	Fine-scale, Pits
VN total length[1] (km)	7701.2	194.1	2571.1
Dominant VN Planimetric Form	Dendritic	Single Thread	Dendritic
Regional VN Density[2] (km^{-1})	0.058	0.015	0.042
Highest Stream Order[3]	7	2	4
Highest Individual VN Density[3] (km^{-1})	0.14	N/A[4]	0.49
Largest Single Valley Network Area (km^2)	43,079[3]	111	722
Candidate Paleolakes	One[5]	Three	None

[1]Values are based on a sinusoidal map projection (358° E center longitude) to remove the effects of map distortion.

[2]Regional valley network density is the total valley network length divided by the area of the map unit.

[3]Highest stream order and individual network density for southern N_{hc1} from Hynek et al. (2010) for network located near 3° S, 5° E. In this study, the highest network density values for western and eastern Nhc1 are located near 4.90° N, 7.80° W, and 3.80° N, 1.70° W (Figure 2c), respectively.

[4]For low order stream systems (N≤2) where there are few (and short) tributaries, estimates of the drainage density are difficult to obtain because the area is ill constrained.

[5]Hynek et al. [2015] propose a depression with a chloride signature is a lacustrine site at ~3˚ S, 8.5˚ W, with an inferred spillover outflow channel to the southwest.

Table S2: Source Image and Location for Figures

Figure	Image ID	Approximate Location[1]
2a	CTX G04_019690_1766_XN_03S005W	3.45˚ S, 5.51˚ W
2b	CTX B17_016169_1793_XI_00S358W	1.7˚ S, 1.8˚ E
2c	CTX P03_002390_1840_XI_04N001W	4.5˚ N, 1.2˚ W
2d	CTX B10_013532_1835_XN_03N006W	5.35˚ N, 7.10˚ W
3a[1]	Quickbird MM128226	21.128˚ S, 69.599˚ W
3b[1]	Quickbird MM128226	21.123˚ S, 69.591˚ W
3c[1]	Quickbird MM128226	21.132˚ S, 69.602˚ W
3d[1]	Quickbird MM128226	21.131˚ S, 69.607˚ W
S3a-d	CTX P03_002390_1840_XI_04N001W	4.5˚ N, 1.2˚ W
S4a-d	CTX D21_035566_1822_XN_02N000W	2.47 ˚ N, 0.82 ˚ W
S4c	CRISM HRS0000D731_07_IF174L_TRR3	2.47 ˚ N, 0.82 ˚ W
S5	CTX G23_027404_1861_XN_06N008W	6.38˚ N, 8.36˚ W
S6[1]	Quickbird MM128226	21.134 ˚ S, 69.584˚ W.
S8a[1,2]	Quickbird MM128226	21.123˚ S, 69.591˚ W
S8b	CTX F22_044454_1845_XI_04N000W	2.96˚ N, 0.02˚ E

[1]Quickbird image have resolution of 0.5 m/pixel.
[2]Locations for figures 3, S6 and S8a are for the Earth.
